# Prevalence and clinical significance of *α*-globin gene subtypes in prenatal thalassemia screening: a focus on the -*α*^3.7III^ variant in Sanya, Hainan, China

**DOI:** 10.3389/fped.2026.1934594

**Published:** 2026-09-30

**Authors:** Xiangxiang Xiao, Cuiyun Li, Tian’e Cai, Liting Chen, Xiaoyan Lin, Yuanyuan Wang, Guiyan Yang, Hui Wei, Wei Li, Ying Yu

**Affiliations:** 1 Department of Medical Genetics and Antenatal Diagnosis Center, Hainan Branch, Shanghai Children’s Medical Center, School of Medicine, Shanghai Jiao Tong University, Sanya, China; 2 Key Laboratory of Molecular Medicine for Women and Children of Hainan Province, Hainan Branch, Shanghai Children's Medical Center, School of Medicine, Shanghai Jiao Tong University, Sanya, China

**Keywords:** - *α* 3.7 III, 3.7 kb deletion *α* -thalassemia, hematological phenotype, thalassemia screening, *α* +-thalassemia

## Abstract

**Introduction:**

Alpha-thalassemia is a common genetic disorder in southern China. The -*α*^3.7^ deletion has three subtypes (I, II, III), among which type III has been rarely reported in the Chinese population. This study aimed to investigate the prevalence, genotypic distribution, and hematological phenotypes of the -*α*^3.7III^ subtype in Sanya, Hainan.

**Methods:**

A total of 474 reproductive-age individuals undergoing prenatal thalassemia screening were enrolled from February 2022 to April 2025. Peripheral blood samples were collected, and genetic testing was performed using next-generation sequencing, with multiplex ligation-dependent probe amplification (MLPA) and Sanger sequencing as supplements.

**Results:**

Among 474 subjects, 182 (38.40%) carried thalassemia mutations, including 149 (31.43%) with *α*-thalassemia. The most common *α*-thalassemia genotype was *α*α/-*α*^3.7I^ (9.70%), followed by *α*α/α^Hb Westmead^*α* (6.12%). A total of 38 -*α*^3.7III^ carriers with a detection rate of 8.02% (38/474), 89.50% of whom were of the Hui ethnic group. Hematological analysis showed that -*α*^3.7III^ carriers exhibited typical microcytic hypochromic features, mean corpuscular volume (MCV), and mean corpuscular hemoglobin (MCH) levels were significantly lower in all -*α*^3.7^-related genotypes than in controls (all *P* < 0.001), while hemoglobin (Hb) levels showed no significant difference (*P* = 0.779). Hematological phenotypes were comparable between -*α*^3.7I^ and -*α*^3.7III^ heterozygotes.

**Discussion:**

This study is the first to report a high prevalence of the -*α*^3.7III^ subtype in the Hainan Hui population, enriching the genetic epidemiological data of α-thalassemia in China. Given that this variant may escape detection by conventional gap-PCR, undiagnosed -*α*^3.7III^ carriers are at substantial risk of transmitting this mutation to their offspring; when inherited in combination with other *α*^0^-thalassemia determinants, including --SEA, --FIL, --THAI, and other related genotypes, there is a significant risk of transfusion-dependent Hb H disease in offspring. These findings support the clinical necessity of comprehensive subtype screening and targeted testing in prenatal diagnosis.

## Introduction

Thalassemia was historically endemic in tropical and subtropical regions. Owing to population migration, it has now become a major global public health concern (1, 2). It is a monogenic recessive disorder caused by reduced or absent synthesis of globin chains. Based on the type of globin chain involved, the most significant forms are *α*-thalassemia and *β*-thalassemia. Among its various forms, *α*-thalassemia is the most prevalent worldwide (3, 4). At the population level, *α*-thalassemia has a markedly higher carrier frequency. Approximately 5% of the global population carry *α*-thalassemia variants (3), and in Hainan Province, 83.9% of thalassemia carriers are *α*-thalassemia carriers (5).

The -*α*^3.7^ deletion is one of the most prevalent types of *α*-thalassemia in Hainan. Early molecular work by Higgs et al. (1984) established the foundational three-subtype classification (-*α*^3.7I^, -*α*^3.7II^, and -*α*^3.7III^) based on distinct breakpoints within the Z homology box of the *α*-globin gene cluster, with crossover intervals resolved using restriction mapping and DNA sequencing (6). Hill et al. further extended this framework to population genetics in 1985 and highlighted broad inter-ethnic heterogeneity in subtype profiles across global cohorts (7). This classification system has subsequently been validated and refined at single-base resolution by modern next-generation and third-generation sequencing technologies (8, 9).

Subtype frequencies differ markedly across ethnic groups. In Chinese populations, -*α*^3.7I^ is widely recognized as the predominant subtype, whereas -*α*^3.7II^ and -*α*^3.7III^ have historically been regarded as rare (10). Conventional gap-PCR assays, the mainstream method for routine thalassemia screening, have inherent technical limitations that may lead to underdetection of the -*α*^3.7III^ subtype (11). In ethnically diverse Hainan Province, most prior local epidemiological studies have only reported the overall carrier rate of the -*α*^3.7^ deletion without further subtype stratification (12), leaving the true local prevalence of the -*α*^3.7III^ subtype unclear.

Internationally, the -*α*^3.7III^ subtype attains high allele frequencies in southwestern Pacific and Oceanian island groups (7, 13), whereas frequencies decline toward mainland Southeast Asia (14). Isolated case reports have recently described -*α*^3.7III^ carriers within Chinese cohorts (8, 11), hinting that its true distribution may be underappreciated.

To date, the population prevalence, ethnic distribution and hematological phenotypic characteristics of the -*α*^3^.⁷III subtype remain poorly characterized in Chinese populations. This study systematically investigates the carrier prevalence, genotypic distribution, and hematological phenotypes of the -*α*^3.7III^ subtype among 474 reproductive-age individuals who underwent prenatal thalassemia screening in Sanya, Hainan. Genetic testing was performed via next-generation sequencing (NGS), followed by Sanger sequencing to define breakpoint junctions and multiplex ligation-dependent probe amplification (MLPA) for orthogonal copy-number validation. The findings address the existing knowledge gap concerning this rare subtype in the Chinese population, clarify its epidemiological characteristics in southern China, and provide evidence for the optimization of thalassemia screening protocols and genetic counseling services for multi-ethnic populations.

## Materials and methods

### Study design and participants

This study enrolled 474 individuals (318 females, 156 males) who were consecutively referred by collaborating antenatal care institutions to our center in Sanya, Hainan Province, for thalassemia genetic testing between February 2022 and May 2025. Participants were enrolled under a Sanya-specific first-line screening protocol, in which pregnant women underwent direct genotyping, while their spouses received initial CBC screening at referring institutions. Genetic testing for spouses was triggered only if the pregnant woman tested positive for thalassemia or if the spouse presented with abnormal CBC parameters (e.g., decreased MCV/MCH). Consequently, the 474 subjects were not paired couples, and pre-testing with hemoglobin electrophoresis was not mandated.

### Sample collection and hematological data acquisition

Five milliliters of peripheral venous blood were collected into EDTA-anticoagulated tubes at the referring institutions and transported to our laboratory at 4 °C. Hematological parameters, including red blood cell count (RBC), hemoglobin (Hb), mean corpuscular volume (MCV), and mean corpuscular hemoglobin (MCH), were supported by the referring institutions.

### DNA extraction and quality control

Genomic DNA was extracted from peripheral blood leukocytes using the DNA Extraction Kit (MGI Tech Co., Ltd., Wuhan, China) according to the manufacturer's instructions. DNA concentration was assessed using a Qubit 4.0 Fluorometer (Thermo Fisher Scientific, Waltham, MA, USA).

### Thalassemia genetic testing

Thalassemia mutations were detected using combinatorial probe-anchor synthesis sequencing (cPAS). Targeted libraries were prepared with the *α*- and *β*-Thalassemia Genetic Testing Kit, and sequencing was performed on the DNBSEQ-200 platform using the Universal Sequencing Reagent Kit (BGI Tech Co., Ltd., Wuhan, China). Data were analyzed using the BGI-developed thalassemia analysis pipeline against the GRCh37/hg19 reference genome. For variant calling, the minimum required depth of 50× for non-deletion variants; quality control and data interpretation were conducted strictly according to the manufacturer's protocols. Common deletion mutations (–^SEA^, -*α*^3.7^, -*α*^4.2^) were independently validated by gap-PCR using the *α*/*β*-Thalassemia gap-PCR Kit (BGI Tech Co., Ltd.). All reagents were purchased from BGI Tech Co., Ltd. (Wuhan, China).

Sanger sequencing confirmed atypical deletions via Gap-PCR. Specific primers (P1：CTCCCACCCTCCCCCTCGCCAAGTC; P2: TTCCTACCCTATCCAGCTGCAGAGA) were synthesized by Sangon Biotech. PCR (25 µL) contained 5× Buffer, dNTPs (25 mmol), MgCl₂ (25 mmol), Taq (2.5 U), primers (10 μmol), and template. Cycling conditions: 96 °C 5 min; 35 cycles (98 °C 30 s, 62 °C 30 s, 72 °C 3 min); 72 °C 10 min. Amplicons were verified by agarose gel electrophoresis prior to bidirectional sequencing (ABI 3730XL). Data were analyzed against GenBank NC_000016.10.

MLPA was performed with the SALSA MLPA Probemix P140-C1 HBA kit (MRC Holland, Amsterdam, the Netherlands) following the manufacturer's protocol (version C1-07). The entire experimental process includes DNA denaturation hybridization, ligation, and PCR amplification. The amplification products are subjected to capillary electrophoresis using the ABI 3500Dx gene analyzer (from Thermo Fisher Company). The data is analyzed using Coffalyser. Net (from MRC-Holland Company). The specific interpretation and quality control standards refer to the instructions of the reagent.

### Statistical analysis

Statistical analyses were performed using the IBM SPSS Statistics program (version 26). Nonparametric data with multiple comparisons were analyzed using the Kruskal–Wallis test followed by Holm's step-down Bonferroni correction for adjusted *P* values. All tests were two sided, and *P* values < 0.05 were considered statistically significant.

## Results

### Genotypic test results

Among 474 subjects (Table 1), 182 (38.40%, 182/474) were identified as thalassemia mutation carriers, including 149 (31.43%, 149/474) cases of *α*-thalassemia, 22 (4.64%, 22/474) cases of *β*-thalassemia, 7 cases of *α*-combined *β*-thalassemia, 2 cases of abnormal hemoglobin variants, and 2 cases of combined *α*-thalassemia and abnormal hemoglobin variants.

**Table 1:** The distribution of genotype.

Classification	Genotype	Count	Ratio (%)
*α*-thalassemia	*α*α/-α^3.7I^	46	9.70
	αα/α^Hb Westmead^α	29	6.12
	αα/-α^4.2^	25	5.27
	αα/-α^3.7III^	24	5.06
	-α^3.7I^/-α^3.7III^	5	1.05
	αα/–^SEA^	4	0.84
	-α^3.7I^/-α^3.7I^	3	0.63
	α^Hb Westmead^α/-α^4.2^	3	0.63
	α^Hb Westmead^α/-α^3.7III^	3	0.63
	-α^4.2^/-α^4.2^	3	0.63
	-α^3.7I^/–^SEA^	2	0.42
	-α^3.7III^/-α^4.2^	1	0.21
	α^Hb Westmead^ α/-α^3.7I^	1	0.21
*β*-thalassemia	*β*^−28 (A^ ^>^ ^G) beta+^/β^N^	10	2.11
	β^Codons 41/42 (−TTCT)^/β^N^	7	1.48
	β^3’UTR^ ^+^ ^129(T^ ^>^ ^A)^/β^N^	4	0.84
	β^Codon 17 (A^ ^>^ ^T)^/β^N^	1	0.21
α-composite β-thalassemia	αα/α^Hb Westmead^α, β^Codons 41/42 (−TTCT)^/β^N^	1	0.21
	αα/-α^4.2^, β^Codons 41/42 (−TTCT)^/β^N^	1	0.21
	αα/-α^3.7I^, β^Codons 41/42 (−TTCT)^/β^N^	1	0.21
	αα/-α^3.7III^, β^Codons 41/42 (−TTCT)^/β^N^	4	0.84
abnormal hemoglobin	β^HBB: c.166A^ ^>^ ^C(Met^ ^>^ ^Leu)^/β^N^	2	0.42
α thalassemia-composite abnormal hemoglobin	αα/-α^4.2^, β^HBB: c.166A^ ^>^ ^C(Met^ ^>^ ^Leu)^/β^N^	1	0.21
	-α^3.7III^/-α^4.2^, β^HBB: c.166A^ ^>^ ^C(Met^ ^>^ ^Leu)^/β^N^	1	0.21
Normal	negative	292	61.60
Total		474	100

Thirteen distinct genotypes were detected across the 149 *α*-thalassemia carriers, with deletional variants accounting for the majority. The most prevalent *α*-thalassemia genotype was heterozygous -*α*^3.7I^ deletion (*α*α/-*α*^3.7I^), with a population carrier frequency of 9.70% (46/474). The next most common deletional genotypes were *α*α/-*α*^4.2^ (5.27%, 25/474) and *α*α/-*α*^3.7III^ (5.06%, 24/474), whereas the *α*α/α^Hb Westmead^*α* variant (6.12%, 29/474) represented the most frequent non-deletional *α*-thalassemia allele.

In total, 38 subjects carried the -*α*^3.7III^ allele (including both simple heterozygotes and compound deletion genotypes), corresponding to an overall detection rate of 8.02% (38/474) in the study cohort. Sex distribution analysis identified 15 male and 23 female carriers. Ethnic stratification showed that 89.50% (34/38) of -*α*^3.7III^ carriers were of Hui ethnicity, while 10.50% (4/38) were of Han ethnicity (Table 2). The atypical 3′UTR breakpoint of the -*α*^3.7III^ subtype was confirmed by Sanger sequencing (Supplementary Figure 1) and further validated by MLPA (Supplementary Figure 2).

**Table 2:** Distribution of different genotypes across gender and ethnicity.

Category	Gender		Ethnicity	
Genotype	Female	Male	Hui	Han
αα/-α^3.7III^	16	8	21	3
-α^3.7I^/-α^3.7III^	3	2	4	1
αα/-α^3.7III^, β^Codons 41/42 (−TTCT)^/β^N^	1	3	4	0
α^Hb Westmead^α/-α^3.7III^	1	2	3	0
-α^3.7III^/-α^4.2^	1	0	1	0
-α^3.7III^/-α^4.2^, β^HBB: c.166A^ ^>^ ^C(Met^ ^>^ ^Leu)^/β^N^	1	0	1	0
Total	23	15	34	4

### Proportions of -α^3.7^ α^+^-thalassemia subtypes

Among 182 thalassemia mutation carriers, 91 individuals (50.00%) were identified as carriers of the -*α*^3.7^ *α*^+^-thalassemia mutation (Table 3), including 79 heterozygotes and 12 homozygotes. Among these 91 carriers, 52.75% (48/91) were *α*α/-*α*^3.7I^, 34.07% (31/91) for *α*α/-*α*^3.7III^, 5.49% (5/91) were -*α*^3.7I^/-*α*^3.7III^, 3.30% (3/91) were -*α*^3.7I^/-*α*^3.7I^, 2.20% (2/91) were -*α*^3.7I^/–^SEA^, and 2.20% (2/91) were -*α*^3.7III^/-*α*^4.2^. The proportion of -*α*^3.7III^ carriers was as high as 41.76% (38/91).

**Table 3:** Distribution of the -α^3.7^ mutation.

Category	Genotype	Count	Ratio (%)
-α^3.7^ heterozygotes	αα/-α^3.7I^	48	52.75
	αα/-α^3.7III^	31	34.07
-α^3.7^ homozygotes	-α^3.7I^/-α^3.7III^	5	5.49
	-α^3.7I^/-α^3.7I^	3	3.30
	-α^3.7I^/–^SEA^	2	2.20
	-α^3.7III^/-α^4.2^	2	2.20
Total		91	100

### Hematological parameter analysis of -α^3.7^ α^+^-thalassemia subtypes

The hematological parameters of enrolled participants, including RBC, HGB, MCV and MCH, across different -*α*^3.7^ *α*-thalassemia subtypes and negative controls are presented in Table 4. A total of 370 subjects were enrolled in the present study, including 292 negative individuals and 78 carriers with four -*α*^3.7^ subtype genotypes, namely *α*α/-*α*^3.7I^, *α*α/-*α*^3.7III^, -*α*^3.7I^/-*α*^3.7I^, and -*α*^3.7I^/-*α*^3.7III^. The -*α*^3.7^ thalassemia carriers exhibited higher median RBC values than the negative group (Figure 1), while no obvious difference in HGB levels was observed among all groups (Figure 2). Both MCV and MCH values decreased progressively from negative controls to heterozygous and homozygous subgroups, showing a distinct descending trend (Figures 3, 4).

**Table 4:** Distribution and comparisons of hematological parameters of different -α^3.7^ α-thalassemia subtypes.

Category	Count	Hematological Parameters	Extreme value	Median value	Mean value
Negative	292	RBC (10^12^/L)	3.39–6.51	4.48	4.53 ± 0.54
		HGB (g/L)	68.00–184.00	133.00	133.78 ± 17.27
		MCV (fL)	56.80–108.10	83.75	83.90 ± 6.40
		MCH (pg)	15.70–40.90	30.30	29.79 ± 2.69
αα/-α^3.7I^	46	RBC (10^12^/L)	3.51–6.70	4.99	4.91 ± 0.66
		HGB (g/L)	71.00–162.00	134.00	133.07 ± 21.19
		MCV (fL)	57.60–101.00	79.20	79.17 ± 6.51
		MCH (pg)	17.20–30.30	27.6	27.16 ± 2.34
αα/-α^3.7III^	24	RBC (10^12^/L)	3.60–5.76	4.75	4.80 ± 0.67
		HGB (g/L)	99.00–169.00	129.00	131.13 ± 18.56
		MCV (fL)	66.50–85.60	77.25	78.08 ± 4.48
		MCH (pg)	21.30–30.90	27.55	27.24 ± 2.29
-α^3.7I^/-α^3.7I^	3	RBC (10^12^/L)	5.27–5.59	5.32	5.39 ± 0.17
		HGB (g/L)	117.00–137.00	127.00	127.00 ± 10
		MCV (fL)	67.40–76.90	71.70	72.00 ± 4.76
		MCH (pg)	22.20–24.50	23.90	23.53 ± 1.19
-α^3.7I^/-α^3.7III^	5	RBC (10^12^/L)	4.32–6.40	5.01	5.40 ± 0.90
		HGB (g/L)	118.00–158.00	121.00	133.00 ± 18.26
		MCV (fL)	67.90–79.40	70.60	71.80 ± 4.61
		MCH (pg)	23.00–28.00	24.20	24.80 ± 1.96
Total	370	RBC (10^12^/L)	3.39–6.70	4.54	4.61 ± 0.59
		HGB (g/L)	68.00–184.00	133.00	133.45 ± 17.78
		MCV (fL)	56.80–108.10	82.70	82.70 ± 6.76
		MCH (pg)	15.70–40.90	29.80	29.18 ± 2.88

**Figure 1:**
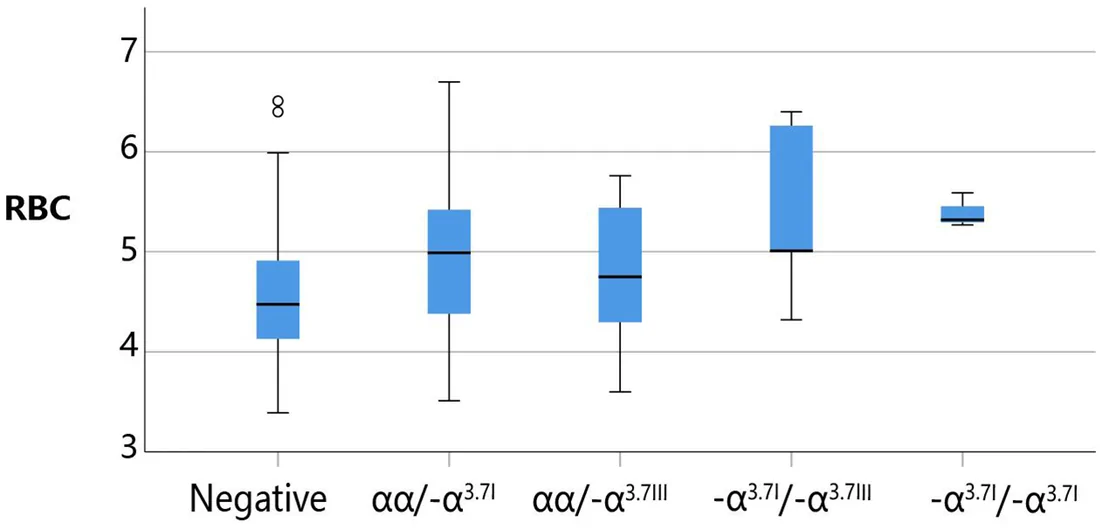
RBC distribution by carrier genotype.

**Figure 2:**
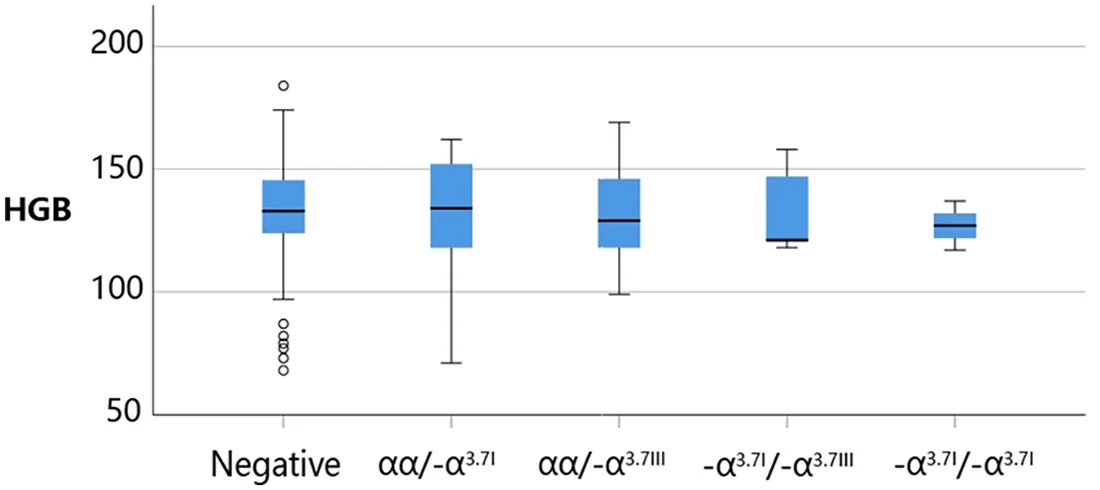
HGB distribution by carrier genotype.

**Figure 3:**
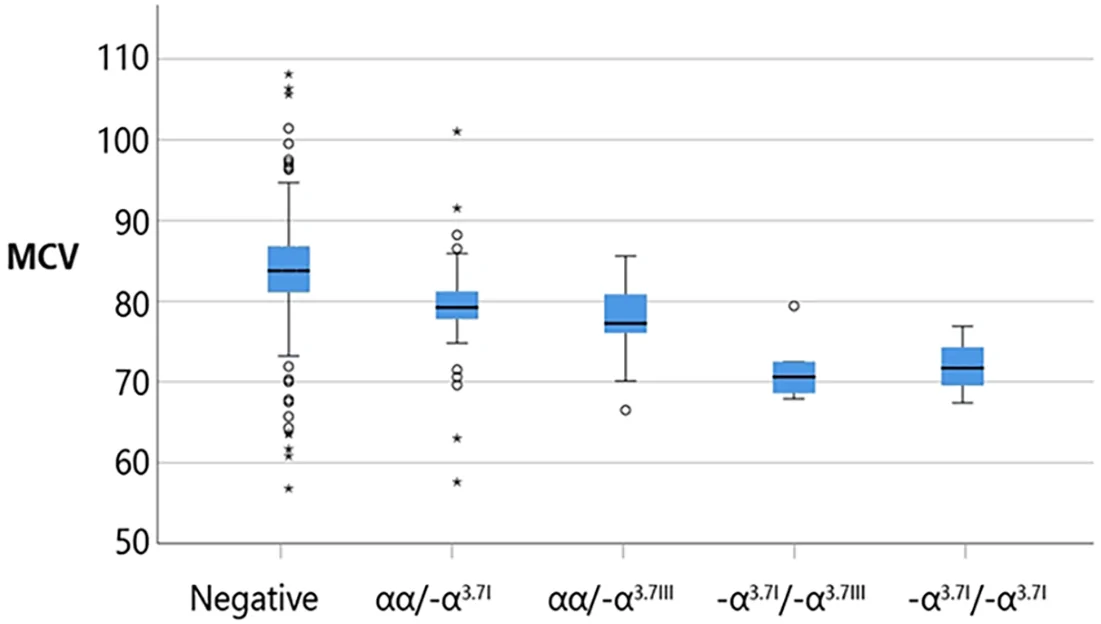
MCV distribution by carrier genotype.

**Figure 4:**
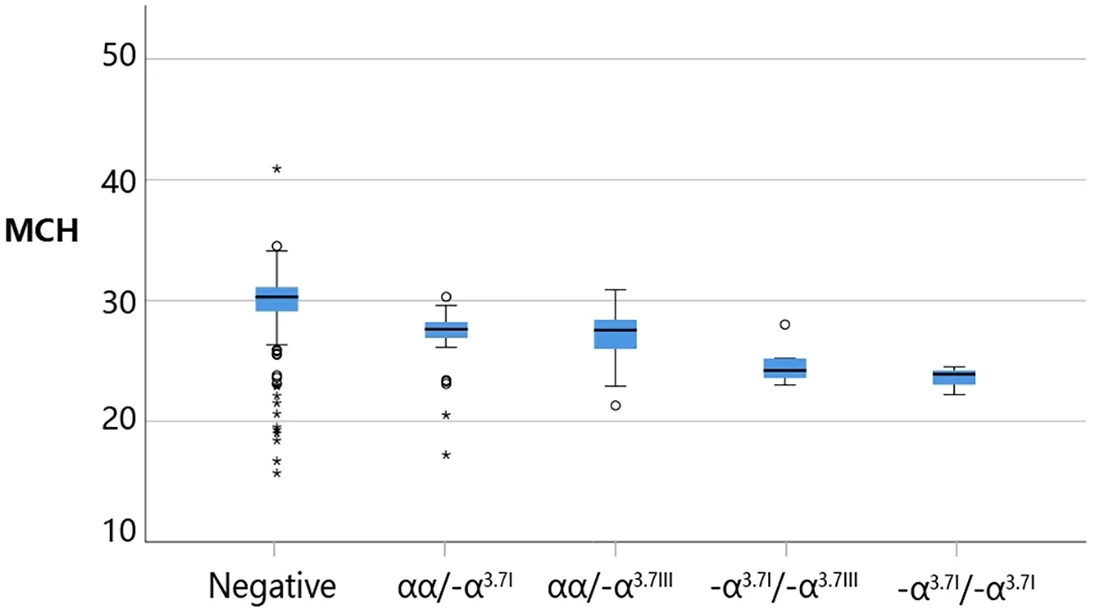
MCH distribution by carrier genotype.

Overall differences in hematological indicators among groups were analyzed using the Kruskal–Wallis test (Table 5). Significant intergroup differences were found in RBC, MCV, and MCH (all *P* < 0.001), whereas no significant difference was observed in Hb levels (*P* = 0.779). Pairwise comparisons with Bonferroni correction were further performed to clarify the specific differences of significant indicators (Supplementary Tables 1–3). For RBC indices, only the difference between the negative group and the *α*α/-*α*^3.7I^ group remained statistically significant after correction (adjusted *P* = 0.001), while no significant differences were detected in other paired comparisons.

**Table 5:** Results of kruskal-wallis test for hematological indices among different genotype groups.

Hematological index	Test statistic (*H* value)	Degree of freedom	*P* value
RBC (×10^12^/L)	27.386	4	0.001
HGB (g/L)	1.763	4	0.779
MCV (fL)	72.382	4	0.001
MCH (pg)	100.061	4	0.001

Kruskal-Wallis test was performed with asymptotic significance (two-sided test) at significance level α=0.05. Test statistics were adjusted for tied ranks.

For MCV and MCH indicators, all -*α*^3.7^-related genotypes showed significantly lower levels compared with the negative group after Bonferroni adjustment. The *α*α/-*α*^3.7I^ and *α*α/-*α*^3.7III^ heterozygous groups presented remarkably decreased MCV and MCH levels (all adjusted *P* < 0.001), and the -*α*^3.7I^/-*α*^3.7I^ and -*α*^3.7I^/-*α*^3.7III^ homozygous and compound heterozygous groups also exhibited significantly reduced hematological parameters (adjusted *P* < 0.05). No significant differences in MCV and MCH were observed either between different heterozygous subtypes or between homozygous/compound heterozygous subtypes.

Collectively, different -*α*^3.7^ *α*-thalassemia subtypes mainly affect erythrocyte volume and intracellular hemoglobin content rather than systemic hemoglobin concentration. The hematological phenotypes gradually aggravated with the severity of gene mutation, presenting the trend of negative group > heterozygous carriers > homozygous/compound heterozygous carriers.

## Discussion

The -*α*^3.7^ deletion is one of the most prevalent *α*^+^-thalassemia variants worldwide, originating from unequal homologous crossover events between the *α*2 and *α*1-globin genes (15). Recombination breakpoints mapping to the X, Y, and Z homologous hotspots differ markedly among the three -*α*^3.7^ subtypes: the -*α*^3.7I^ subtype arises from high-homology regions at the 5′ end of the Z box, the -*α*^3.7II^ subtype involves a 7-bp insertion in IVSII of the *α*1 gene, whereas the rare -*α*^3.7III^ subtype is characterized by recombination within a conserved 46-bp sequence in the 3′ untranslated region (3′UTR) of the *HBA2* gene (14). Such breakpoint heterogeneity potentially alters the regulatory function of the fused residual *α*-globin gene, and subtyping of -*α*^3.7^ deletion is essential for elucidating the evolutionary characteristics of the *α*-globin gene cluster.

In the present study, a total of 38 -*α*^3.7III^ carriers were identified among 474 reproductive-age individuals, yielding a detection rate of 8.02%. This finding deviates notably from the prevailing understanding of *α*-thalassemia subtype distribution in southern China. To date, most population-based studies in Hainan Province, including those conducted in Li, Miao and Han ethnic groups, have only reported the overall frequency of the -*α*^3.7^ deletion without subtype stratification, and none have documented a high prevalence of the -*α*^3.7III^ subtype (12). In broader southern Chinese populations such as Guangdong and Guangxi, -*α*^3.7I^ has been consistently recognized as the predominant subtype (10), while -*α*^3.7III^ has only been reported as sporadic cases in individual rare variant studies (8, 11). Compared with these limited case reports, the relatively high prevalence observed in our cohort challenges the perception that -*α*^3.7III^ is universally rare across Chinese populations, and confirms that this variant presents striking regional and ethnic aggregation rather than uniform low frequency.

The discrepancy between our findings and prior domestic reports can be attributed to two major factors. First, methodological limitations: the 3′UTR breakpoint of the -*α*^3.7III^ subtype falls outside the amplicon range of most conventional commercial gap-PCR kits, making this variant prone to underdetection in routine screening. The lack of standardized subtype-specific detection protocols has further contributed to its long-term underestimation in Chinese populations. Second, differences in study population composition: most prior studies recruited predominantly Han Chinese participants, whereas our cohort includes a substantial proportion of the Sanya Hui (Utsul) population, a unique ethnic group with distinct ancestral origins.

From a global perspective, the distribution of the -*α*^3.7III^ subtype exhibits pronounced geographical and ethnic stratification, forming a clear frequency gradient across regions. At one end of the spectrum, the variant shows high prevalence in Oceanian populations: the estimated population carrier rates are approximately 21.7% in Vanuatu and 23.0% in French Polynesian islanders, converted from the reported allele frequencies of 11.5% and 7.5% respectively based on Hardy-Weinberg equilibrium, and it represents the dominant form of the -*α*^3.7^ deletion in these regions (7, 13). In Southeast Asia, the population carrier rate is markedly lower and geographically variable. A population-based screening in northeastern Thailand documented a directly measured carrier rate of 0.17% (14), data from southern Thailand is limited to patient-level cohorts, with a subtype detection rate of 2.31% among 260 Hb H disease cases (16). In contrast, the -*α*^3.7III^ subtype is extremely rare or absent in Mediterranean, African, where -*α*^3.7I^ accounts for nearly all -*α*^3.7^ deletions (6). The 8.02% prevalence observed in our Sanya cohort is markedly higher than that in mainland Southeast Asian populations and close to the level seen in Pacific Islander groups, situating the study population at an intermediate position in this global epidemiological gradient.

This distinctive global distribution pattern is shaped by multiple evolutionary and demographic mechanisms. The high frequency in Oceanian populations is thought to reflect a combination of founder effect and genetic drift during the initial peopling of remote Pacific islands, coupled with potential positive selection under historical malaria endemicity (17). The moderate prevalence in Southeast Asia aligns with the region's role as an intermediate node of Austronesian migration. For the Sanya Hui population, the high frequency observed in this study is most consistent with a founder effect introduced by ancestral migrants from Champa (present-day central Vietnam), followed by further enrichment due to long-term relative geographic and cultural isolation of the settlement community. This population history explains why the variant is highly enriched in this specific ethnic group but remains rare in the broader Han Chinese population.

Ethnic stratification analysis in our cohort further supports this demographic interpretation. Among the 38 -*α*^3.7III^ carriers, 89.50% (34/38) were of Hui ethnicity (Utsul) and only 10.50% (4/38) were of Han ethnicity, indicating a strong genetic association between the -*α*^3.7III^ variant and the Sanya Hui population. Historical and anthropological evidence indicates that the Sanya Hui originated from the ancient Champa kingdom and migrated to Hainan during the Song and Yuan dynasties, representing descendants of Southeast Asian Muslim populations along the Maritime Silk Road (18). These findings provide direct molecular evidence supporting the Southeast Asian origin hypothesis of the Sanya Hui population, and illustrate how historical migration events along the Maritime Silk Road have shaped the genetic structure of coastal populations in southern China.

The small number of Han Chinese -*α*^3.7III^ carriers detected in this cohort can be explained by two complementary mechanisms. First, long-term interethnic marriage and residential admixture between Hui and Han populations have resulted in genetic introgression, representing normal bidirectional gene flow between neighboring ethnic groups. Second, the continuous improvement of molecular diagnostic technologies, including long-read sequencing and high-resolution targeted PCR assays, has enhanced detection sensitivity, enabling the identification of low-frequency carriers that were previously missed by conventional screening methods.

Hematological phenotypic analysis showed that -*α*^3.7III^ heterozygous carriers presented typical microcytic hypochromic features, with significantly reduced MCV and MCH levels. The Kruskal–Wallis test revealed significant overall differences in RBC, MCV and MCH levels across different -*α*^3.7^ genotype groups (all *P* < 0.001), whereas no significant difference in HGB levels was observed among groups (*P* = 0.779). *Post-hoc* pairwise comparisons showed no statistically significant differences in RBC, MCV and MCH between heterozygous -*α*^3.7I^ and -*α*^3.7III^ carriers, consistent with previously reported mild phenotypic features of -*α*^3.7III^ heterozygotes (11). This finding indicates that the two heterozygous variants exert comparable effects on hematological parameters, suggesting that separate identification of these two subtypes is not required for routine clinical risk stratification.

A clear genotype-phenotype dose-response relationship was observed across genotype groups, with hematological parameter severity graded as follows: negative control group > heterozygous groups (*α*α/-*α*^3.7I^, *α*α/-*α*^3.7III^) > homozygous and compound heterozygous groups (-*α*^3.7I^/-*α*^3.7I^, -*α*^3.7I^/-*α*^3.7III^). Homozygous and compound heterozygous carriers had significantly lower MCV and MCH levels than negative controls, confirming that the severity of erythrocyte morphological abnormalities is positively correlated with the number of deleted *α*-globin gene copies (19–21). This study further validates that MCV and MCH are sensitive and reliable hematological biomarkers for *α*⁺-thalassemia screening (22, 23). In addition, the stable HGB levels across all genotype groups indicate that all -*α*^3.7^ subtype carriers, regardless of zygosity, present only mild erythrocyte morphological changes without overt clinical anemia. These silent or mild phenotypic features can be easily confused with iron deficiency anemia, underscoring the necessity of genetic testing for definitive diagnosis.

## Conclusion

Given that the 3′UTR breakpoint of the -*α*^3.7III^ subtype lies outside the target amplification region of most conventional gap-PCR assays, this variant may be susceptible to underdetection in routine thalassemia screening. This potential diagnostic gap carries clinical implications for reproductive risk assessment: undiagnosed -*α*^3.7III^ carriers who partner with carriers of *α*^0^-thalassemia determinants, including -SEA, -FIL, -THAI, and other related genotypes, would have a significant risk of having offspring with transfusion-dependent Hb H disease, a risk that may be underestimated under current standard screening protocols.

Accordingly, in high-prevalence thalassemia regions of southern China, targeted screening for the -*α*^3.7III^ subtype may be considered for routine preconception and prenatal care, particularly for ethnic populations with documented Southeast Asian genetic ancestry such as the Sanya Hui. For individuals with unexplained microcytic hypochromic anemia (MCV < 80 fL, MCH < 27 pg) and negative results on conventional gap-PCR testing, secondary analysis for rare deletions is recommended. Advanced molecular methods, including 3′UTR-targeted PCR, multiplex ligation-dependent probe amplification and long-read sequencing, can be adopted to reduce missed diagnoses. Optimized screening strategies will support more accurate genetic counseling, facilitate targeted clinical intervention, and ultimately contribute to the prevention of severe thalassemia in newborns.

## Limitations

This single-center study focused on the Sanya Hui population, limiting the generalizability of the findings to broader Chinese regions and ethnic groups; additionally, it only analyzed the prevalence and hematological phenotypes of the -*α*^3.7III^ subtype without in-depth molecular mechanism exploration, family-based validation, or screening strategy comparison. Future studies will adopt a multi-center, multi-ethnic design with expanded sample sizes, conduct functional verification of the variant, optimize targeted screening protocols for rare thalassemia subtypes, perform family risk assessment, and establish a shared database to improve the precision of prenatal diagnosis and genetic counseling for *α*-thalassemia.

## Data Availability

The datasets presented in this article are not readily available due to patient privacy and ethical restrictions and these data cannot be deposited into a public repository. Requests to access the datasets should be directed to the corresponding author.
