# Supplementary material for: Prevalence and clinical significance of α-globin gene subtypes in prenatal thalassemia screening: a focus on the -α3.7III variant in Sanya, Hainan, China
Source: Front Pediatr. 2026 Sep 30;14:1934594. doi: 10.3389/fped.2026.1934594 (PMC13661913; doi:10.3389/fped.2026.1934594)
Supplement: Supplementary file 1 [file Supplementaryfile1.docx]

Supplementary Material

# 1. Supplementary Figures

Supplementary Figure S1. Sanger sequencing result of -α^3.7III^ subtypes.

The PCR products were subjected to verify the breakpoints. The result showed that the breakpoints were the same as literature reports.

Supplementary Figure S2. MLPA of -α^3.7^ subtypes.

(A) -α^3.7I^ subtypes : MLPA probe-ratio plot showing a ~0.5 dosage ratio for *HBA2*-specific probes (red arrow) while *HBA1*-specific probes retain a normal ~1.0 ratio (blue arrow), indicating the loss of the *HBA2* gene. NGS coverage shows a sharp drop in the *HBA2* region. This subtype is readily detected by standard Gap-PCR.

(B) -α^3.7III^ subtypes: MLPA probe-ratio plot displaying a ~0.5 dosage ratio for *HBA1*-specific probes (blue arrow) but a normal ~1.0 ratio for *HBA2*-specific probes (red arrow), confirming the loss of the *HBA1* gene. NGS coverage reveals reduced read depth over the *HBA1* body and partial dropout of the *HBA2* 3’UTR. This subtype is missed by conventional Gap-PCR due to primer mismatch.

(C) Compound heterozygous -α^3.7I^/-α^3.7III^ genotype: MLPA probe-ratio plot demonstrating a composite pattern where both *HBA1*-specific and *HBA2*-specific probes show reduced ~0.5 ratios, while probes targeting shared alpha-globin sequences retain a ~1.0 ratio. This unique “double-drop” fingerprint accurately resolves the complex trans arrangement that is indistinguishable by standard assays.

# 2. Supplementary Tables

Table 1 Post Hoc Pairwise Comparisons for Simplified RBC Indices

| Sample1-Sample 2 | Test Statistic | Standard Error | Standardized Test Statistic | *P* value | Adjusted *P* value^a^ |
| --- | --- | --- | --- | --- | --- |
| Negative-αα/-α^3.7I^ | -64.974 | 16.966 | -3.830 | 0.000 | 0.001 |
| Negative-αα/-α^3.7III^ | -48.443 | 22.711 | -2.133 | 0.033 | 0.329 |
| Negative--α^3.7I^/-α^3.7III^ | -109.885 | 48.237 | -2.278 | 0.023 | 0.227 |
| Negative--α^3.7I^/-α^3.7I^ | -157.818 | 62.064 | -2.543 | 0.011 | 0.110 |
| αα/-α^3.7III^ -αα/-α^3.7I^ | 16.531 | 26.931 | 0.614 | 0.539 | 1.000 |
| αα/-α^3.7III^ --α^3.7I^/-α^3.7III^ | -61.442 | 52.576 | -1.169 | 0.243 | 1.000 |
| αα/-α^3.7III^--α^3.7I^/-α^3.7I^ | -109.375 | 65.493 | -1.670 | 0.095 | 0.949 |
| αα/-α^3.7I^ --α^3.7I^/-α^3.7III^ | -44.911 | 50.362 | -0.892 | 0.373 | 1.000 |
| αα/-α^3.7I^ --α^3.7I^/-α^3.7I^ | -92.844 | 63.730 | -1.457 | 0.145 | 1.000 |
| -α^3.7I^/-α^3.7III^ --α^3.7I^/-α^3.7I^ | -47.933 | 78.105 | -0.614 | 0.539 | 1.000 |

Note：^a^The significance level for multiple comparisons was adjusted using the Bonferroni correction method.

Table 2 Post Hoc Pairwise Comparisons for Simplified MCV Indices

| Sample1-Sample 2 | Test Statistic | Standard Error | Standardized Test Statistic | *P* value | Adjusted *P* value^a^ |
| --- | --- | --- | --- | --- | --- |
| -α^3.7I^/-α^3.7I^--α^3.7I^/-α^3.7III^ | 4.867 | 78.105 | 0.062 | 0.950 | 1.000 |
| -α^3.7I^/-α^3.7I^--αα/-α^3.7III^ | 64.854 | 65.493 | 0.990 | 0.322 | 1.000 |
| -α^3.7I^/-α^3.7I^--αα/-α^3.7I^ | 82.819 | 63.729 | 1.300 | 0.194 | 1.000 |
| -α^3.7I^/-α^3.7I^--Negative | 181.956 | 62.064 | 2.932 | 0.003 | 0.034 |
| -α^3.7I^/-α^3.7III^--αα/-α^3.7III^ | 59.988 | 52.576 | 1.141 | 0.254 | 1.000 |
| -α^3.7I^/-α^3.7III^--αα/-α^3.7I^ | 77.952 | 50.362 | 1.548 | 0.122 | 1.000 |
| -α^3.7I^/-α^3.7III^-Negative | 177.089 | 48.237 | 3.671 | 0.000 | 0.002 |
| αα/-α^3.7III^-αα/-α^3.7I^ | 17.965 | 26.931 | 0.667 | 0.505 | 1.000 |
| αα/-α^3.7III^ -Negative | 117.102 | 22.711 | 5.156 | 0.000 | 0.000 |
| αα/-α^3.7I^-Negative | 99.137 | 16.966 | 5.843 | 0.000 | 0.000 |

Note：^a^The significance level for multiple comparisons was adjusted using the Bonferroni correction method.

Table 3 Post Hoc Pairwise Comparisons for Simplified MCH Indices

| Sample1-Sample 2 | Test Statistic | Standard Error | Standardized Test Statistic | *P* value | Adjusted *P* value^a^ |
| --- | --- | --- | --- | --- | --- |
| -α^3.7I^/-α^3.7I^--α^3.7I^/-α^3.7III^ | 14.233 | 78.095 | 0.182 | 0.855 | 1.000 |
| -α^3.7I^/-α^3.7I^-αα/-α^3.7I^ | 59.225 | 63.721 | 0.929 | 0.353 | 1.000 |
| -α^3.7I^/-α^3.7I^-αα/-α^3.7III^ | 69.833 | 65.484 | 1.066 | 0.286 | 1.000 |
| -α^3.7I^/-α^3.7I^-Negative | 192.284 | 62.056 | 3.099 | 0.002 | 0.019 |
| -α^3.7I^/-α^3.7III^-αα/-α^3.7I^ | 44.991 | 50.355 | 0.893 | 0.372 | 1.000 |
| -α^3.7I^/-α^3.7III^-αα/-α^3.7III^ | 55.6 | 52.569 | 1.058 | 0.290 | 1.000 |
| -α^3.7I^/-α^3.7III^-Negative | 178.05 | 48.231 | 3.692 | 0.000 | 0.002 |
| αα/-α^3.7I^-αα/-α^3.7III^ | -10.609 | 26.927 | -0.394 | 0.694 | 1.000 |
| αα/-α^3.7I^-Negative | 133.059 | 16.963 | 7.844 | 0.000 | 0.000 |
| αα/-α^3.7III^-Negative | 122.45 | 22.707 | 5.393 | 0.000 | 0.000 |

Note：^a^The significance level for multiple comparisons was adjusted using the Bonferroni correction method.
